# Association between nontraditional lipid profiles and peripheral arterial disease in Chinese adults with hypertension

**DOI:** 10.1186/s12944-020-01407-3

**Published:** 2020-11-03

**Authors:** Congcong Ding, Yang Chen, Yumeng Shi, Minghui Li, Lihua Hu, Wei Zhou, Tao Wang, Lingjuan Zhu, Xiao Huang, Huihui Bao, Xiaoshu Cheng

**Affiliations:** 1 Department of Cardiovascular Medicine, the Second Affiliated Hospital of Nanchang University, No. 1 Minde Road, Nanchang, 330006 Jiangxi Province China; 2 Center for Prevention and Treatment of Cardiovascular Diseases, the Second Affiliated Hospital of Nanchang University, Nanchang, Jiangxi Province China

**Keywords:** Nontraditional lipid profiles, Peripheral artery disease, Hypertension, Ankle-brachial index, Cholesterol, Chinese adults, Epidemiology

## Abstract

**Background:**

Data on the relationship between nontraditional lipid profiles [total cholesterol (TC)/high-density lipoprotein cholesterol (HDL-C) ratio, triglyceride (TG)/HDL-C ratio, low-density lipoprotein cholesterol (LDL-C)/HDL-C ratio, non-high-density lipoprotein cholesterol (non-HDL-C)] and the risk of peripheral artery disease (PAD) are limited. The present study investigated the relationship of nontraditional lipid indices with PAD in hypertensive patients.

**Methods:**

This cross-sectional study was performed among 10,900 adults with hypertension. Participants were diagnosed with PAD when their ankle-brachial index (ABI) was < 0.9. The association between nontraditional lipid profiles and PAD was examined using multivariate logistic regression analysis and the restricted cubic spline.

**Results:**

All nontraditional lipid indices were independently and positively associated with PAD in a dose-response fashion. After multivariable adjustment, the per SD increments of the TC/HDL-C, TG/HDL-C, LDL-C/HDL-C ratios and non-HDL-C were all significantly associated with 37, 14, 40, and 24% higher risk for PAD, respectively. The adjusted ORs (95% CI) for PAD were 1.77 (1.31, 2.40), 1.71 (1.25, 2.34), 2.03 (1.50, 2.74), and 1.70 (1.25, 2.31) when comparing the highest tertile to the lowest tertile of the TC/HDL-C, TG/HDL-C, LDL-C/HDL-C ratios and non-HDL-C, respectively.

**Conclusions:**

Among Chinese hypertensive adults, all nontraditional lipid indices were positively associated with PAD, and the LDL-C/HDL-C and TC/HDL-C ratios were better than the other nontraditional lipid indices for predicting PAD. These findings may improve the risk stratification of cardiovascular disease and dyslipidemia management.

**Trial registration:**

CHiCTR, ChiCTR1800017274. Registered 20 July 2018.

**Supplementary Information:**

**Supplementary information** accompanies this paper at 10.1186/s12944-020-01407-3.

## Background

Peripheral artery disease (PAD) is a clinical manifestation of systemic atherosclerosis. PAD is estimated to affect 200 million people globally, and it is a strong predictor of cardiovascular causes of death [1]. However, PAD is not fully understood and treated [2, 3]. The ankle-brachial index (ABI), which is commonly known as the ratio of the systolic blood pressure (SBP) measured at the ankle and arm, is a reliable, inexpensive, and noninvasive test for the diagnosis of PAD [4]. However, more than half of patients with PAD are asymptomatic, and an absence of routine ABI measurement in the primary care setting exists in most countries. Agents that reliably indicate subclinical atherosclerosis for patients who are at high risk of suffering from PAD are urgently needed.

Traditional lipid parameters, such as abnormal rates of high-density lipoprotein cholesterol (HDL-C) and low-density lipoprotein cholesterol (LDL-C), are common risk factors for PAD [5]. Notably, an increasing number of studies have indicated the development and progression of atherosclerosis under conditions of abnormal nontraditional lipid profiles. The triglyceride (TG)/HDL-C ratio may be a favourable predictor of cardiovascular disorders [6–9], and many studies have suggested that the total cholesterol (TC)/HDL-C ratio is also closely related to cardiovascular disease (CVD) [10–12]. Growing evidence has validated the relationship between the LDL-C/HDL-C ratio and the carotid plaque score [13], carotid intima media thickness [14], and PAD [12]. In addition, non-high-density lipoprotein cholesterol (non-HDL-C), which is the sum of cholesterol in lipoproteins other than HDL-C, was also shown to be a better predictor for major adverse cardiovascular events than LDL-C in a prospective observation study [15].

Increasing research shows a strong link between hypertension and PAD [16]. Therefore, the identification of lipid-related risk factors in people with hypertension will help improve strategies for population-based screening and prevention in subjects who are at risk for vascular complications. However, few studies have investigated the association between all nontraditional lipid indices and PAD risk. No previous study has examined this association in a population with hypertension. The present study comprehensively determined the association between nontraditional lipid profiles and PAD and identified the best surrogate indicators for predicting the risk of PAD risk in Chinese adults with hypertension.

## Methods

### Subject population and design

The subject population in this study was selected from the ongoing China Hypertension Registry Study (registration number: ChiCTR1800017274), a large-scale, real-world observational registry study. It was designed to determine the prevalence and control of hypertension and evaluate the prognostic factors of hypertension from this registry of hypertension in rural areas of southern China. All study subjects were hypertension patients who were at least 18 years old. Eligible participants had at least one of the following factors: (1) individuals with an SBP > 140 mmHg or/and diastolic blood pressure (DBP) ≥ 90 mmHg in the relaxed sitting position at the initial screening; (2) individuals receiving antihypertensive medications; or (3) individuals with a history of hypertension. Participants with neurological abnormalities or who were unable to complete the follow-up visit according to the research protocol were excluded from the study. The ethics committee of the Institute of Biomedicine, Anhui Medical University, approved the study. All study subjects signed a written informed consent before their enrolment in this study.

**Fig. 1:**
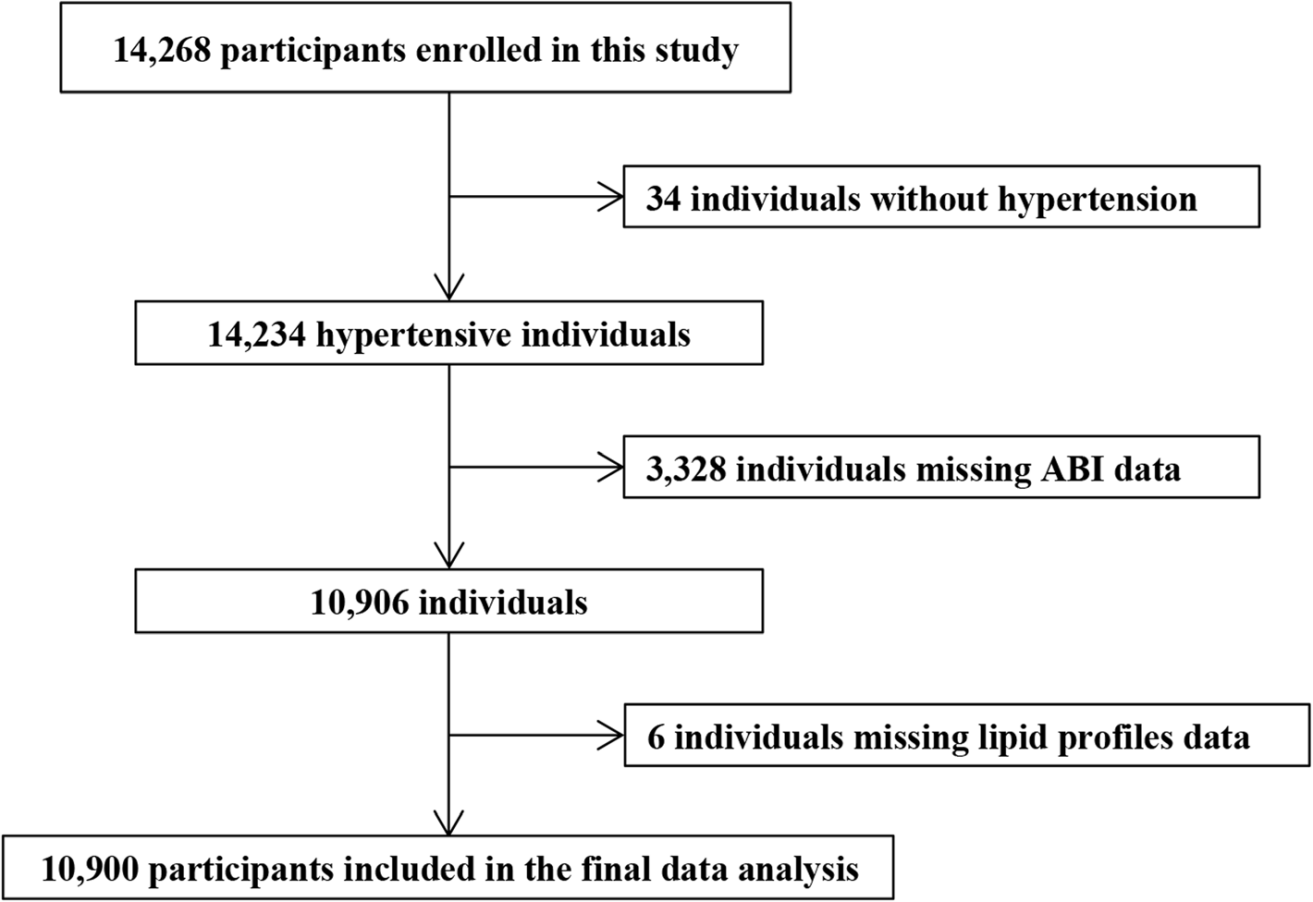
Flow chart of participants

Ultimately, the present study recruited 14,268 eligible study subjects in Wuyuan County in Jiangxi Province, China from March 2018 to August 2018. After excluding subjects without hypertension (*n* = 34) and subjects with missing ABI (*n* = 3328) and lipid profile values (*n* = 6), a total of 10,900 hypertensive patients were included in the final analyses (Fig. 1).

### Data collection

The research staff were strictly trained to perform health interviews for the study population. A questionnaire was used to collect demographic information including age, sex, lifestyle data (e.g., alcohol drinking status and smoking status), medical history and medication information. The anthropometric measurement indicators included weight and height. Blood pressure (BP) was obtained using an electronic sphygmomanometer (Omron; Dalian, China) after the individual rested in a seated position for more than 5 min. Three measurements on the right arm were performed with one-minute intervals between successive readings, and the mean value was calculated.

After more than 12 h of overnight fasting, all venous blood samples of the study participants were collected, frozen and transported to Biaojia Biotechnology in Shenzhen in Guangdong Province, China. Automatic clinical analysers (Beckman Coulter, USA) were used to measure serum fasting glucose, lipids (HDL-C, LDL-C, TG and TC), creatinine and total homocysteine (tHcy). All laboratory operations complied with the standardization and certification procedures.

The TC/HDL-C, TG/HDL-C, and LDL-C/HDL-C ratios were calculated as TC, TG, and LDL-C divided by HDL-C, respectively. Non-HDL-C was calculated as HDL-C subtracted from TC. The body mass index (BMI) was calculated by dividing the weight by the height squared. The estimated glomerular filtration rate (eGFR) was calculated using the formula derived from the Chronic Kidney Disease Epidemiology Collaboration [17].

### Pad

Participants were diagnosed with PAD when an ABI < 0.9 existed in either leg [18]. According to standard clinical guidelines, the ABI was automatically measured with the individuals lying flat on their back [19]. Measurements were performed after the participants had rested for at least 10 min using the BP-203RPE III device (Omron, Kyoto, Japan). The ABI was calculated as the highest SBP at the ankles divided by the highest SBP of the right or left upper arms. The lower ABI values calculated for the left and right ankles were used in the analysis.

### Covariables

The covariates were selected based on their clinical importance, statistical significance in the univariable analysis of their associations with PAD, and the potential confounder effect estimates individually changing by at least 10%. The covariates were age, sex, BMI, SBP, DBP, smoking status, alcohol drinking status, fasting glucose, tHcy, eGFR, self-reported diabetes, self-reported stroke, use of lipoprotein-lowering drugs, and use of antihypertensive drugs.

### Statistical analyses

The mean ± SD or the median (25th percentile, 75th percentile) and number (%) were used to describe continuous data and categorical data, respectively. Differences in data characteristics with or without PAD were compared using Student’s t-test for normally distributed data, nonparametric Mann-Whitney test for abnormally distributed data, and a chi-squared test for categorical data. Logistic regression analyses were performed to assess the association between nontraditional lipid indices (the non-HDL-C, LDL-C/HDL-C, TG/HDL-C and TC/HDL-C ratios) and PAD by presenting the odds ratio (OR) and 95% confidence interval (CI) with adjustment for pertinent variables. Nontraditional lipid profiles were assessed by establishing models for both continuous data per SD increment and categorical data using tertiles with the lowest tertile (T1) as the reference group. Trend tests were calculated using the nontraditional lipid profile tertile categories as continuous data. Restricted cubic spline (smooth fitting curve) was performed to visually evaluate the association with nontraditional lipid profiles of PAD. Potential effect modifiers were evaluated by subgroup and interaction analyses.

All the data analyses were performed using R version 3.4.3 (www.R-project.org) and EmpowerStates (www.empowerstats.com). A 2-sided *P* < 0.05 was considered statistically significant.

## Results

### Baseline characteristics

The final data analyses included 10,900 patients with hypertension. Table 1 presents the participant characteristics for all subjects and subjects with and without PAD. Overall, the mean age was 63.9 years and 47.1% of participants were males. Compared with the participants without PAD, those with PAD tended to have a higher age, SBP, tHcy, TC/HDL-C ratio, and LDL-C/HDL-C ratio; be male and a current smoker; have a higher rate of self-reported stroke and antihypertensive or antiplatelet drug use; and have a lower BMI, DBP, eGFR and HDL-C level (all *P* < 0.05). No significant differences were found between participants with or without PAD in terms of fasting glucose, LDL-C, TG, TC, TG/HDL-C ratio, non-HDL-C level, current alcohol drinking, self-reported diabetes, or use of glucose-lowering drugs or lipoprotein-lowering drugs.

**Table 1:** Characteristics of study participants with or without PAD

Variables	Total (***n*** = 10,900)	non-PAD (***n*** = 10,590)	PAD (***n*** = 310)	***P*** value
Age, years	63.9 ± 9.3	63.6 ± 9.1	71.6 ± 10.0	< 0.001
Male, n (%)	5129 (47.1%)	4958 (46.8%)	171 (55.2%)	0.004
BMI, kg/m^2^	23.6 ± 3.8	23.6 ± 3.8	22.5 ± 3.9	< 0.001
SBP, mm Hg	148.5 ± 18.2	148.4 ± 18.0	152.8 ± 22.7	< 0.001
DBP, mm Hg	89.1 ± 11.5	89.2 ± 11.5	84.1 ± 11.5	< 0.001
Current smoking, n (%)	2868 (26.3%)	2745 (25.9%)	123 (39.7%)	< 0.001
Current alcohol drinking, n (%)	2469 (22.7%)	2409 (22.8%)	60 (19.4%)	0.159
Self-reported diabetes, n (%)	1176 (10.8%)	1145 (10.8%)	31 (10.0%)	0.650
Self-reported stroke, n (%)	708 (6.5%)	667 (6.3%)	41 (13.2%)	< 0.001
**Laboratory results**				
Fasting glucose, mmol/L	6.2 ± 1.6	6.2 ± 1.6	6.1 ± 1.5	0.439
Total homocysteine, μmol/L	18.0 ± 11.0	17.9 ± 10.8	22.5 ± 17.1	< 0.001
eGFR, mL · min^− 1^ · 1.73 m^− 2^	88.7 ± 20.4	89.1 ± 20.1	74.8 ± 23.3	< 0.001
TC, mmol/L	5.1 ± 1.1	5.1 ± 1.1	5.1 ± 1.2	0.966
TG, mmol/L	1.4 (1.0–2.1)	1.4 (1.0–2.1)	1.4 (1.0–2.1)	0.069
LDL-C, mmol/L	3.0 ± 0.8	3.0 ± 0.8	3.1 ± 0.9	0.156
HDL-C, mmol/L	1.6 ± 0.4	1.6 ± 0.4	1.5 ± 0.4	0.001
TC/HDL-C ratio	3.4 ± 0.8	3.4 ± 0.8	3.5 ± 0.9	< 0.001
TG/HDL-C ratio	0.9 (0.6–1.5)	0.9 (0.6–1.5)	0.9 (0.6–1.5)	0.304
LDL-C/HDL-C ratio	2.0 ± 0.7	2.0 ± 0.6	2.1 ± 0.7	< 0.001
Non-HDL-C, mmol/L	3.6 ± 1.0	3.6 ± 1.0	3.6 ± 1.1	0.163
**Medication use, n (%)**				
Antihypertensive drugs	7155 (65.6%)	6930 (65.4%)	225 (72.6%)	0.009
Glucose-lowering drugs	572 (5.2%)	554 (5.2%)	18 (5.8%)	0.654
Lipoprotein-lowering drugs	381 (3.5%)	369 (3.5%)	12 (3.9%)	0.715
Antiplatelet drugs	429 (3.9%)	409 (3.9%)	20 (6.5%)	0.021

Data are expressed as mean ± standard deviation or median (interquartile range) and numbers (percentage) as appropriate

Abbreviations: *PAD* Peripheral arterial disease; *BMI* Body mass index; *SBP* Systolic blood pressure; *DBP* Diastolic blood pressure; *eGFR* Estimated glomerular filtration rate; *TC* Total cholesterol; *TG* Triglyceride; *HDL-C* High-density lipoprotein cholesterol; *LDL-C* Low-density lipoprotein cholesterol; *non-HDL-C* Non-high-density lipoprotein cholesterol

### Nontraditional lipid profiles and PAD

**Fig. 2:**
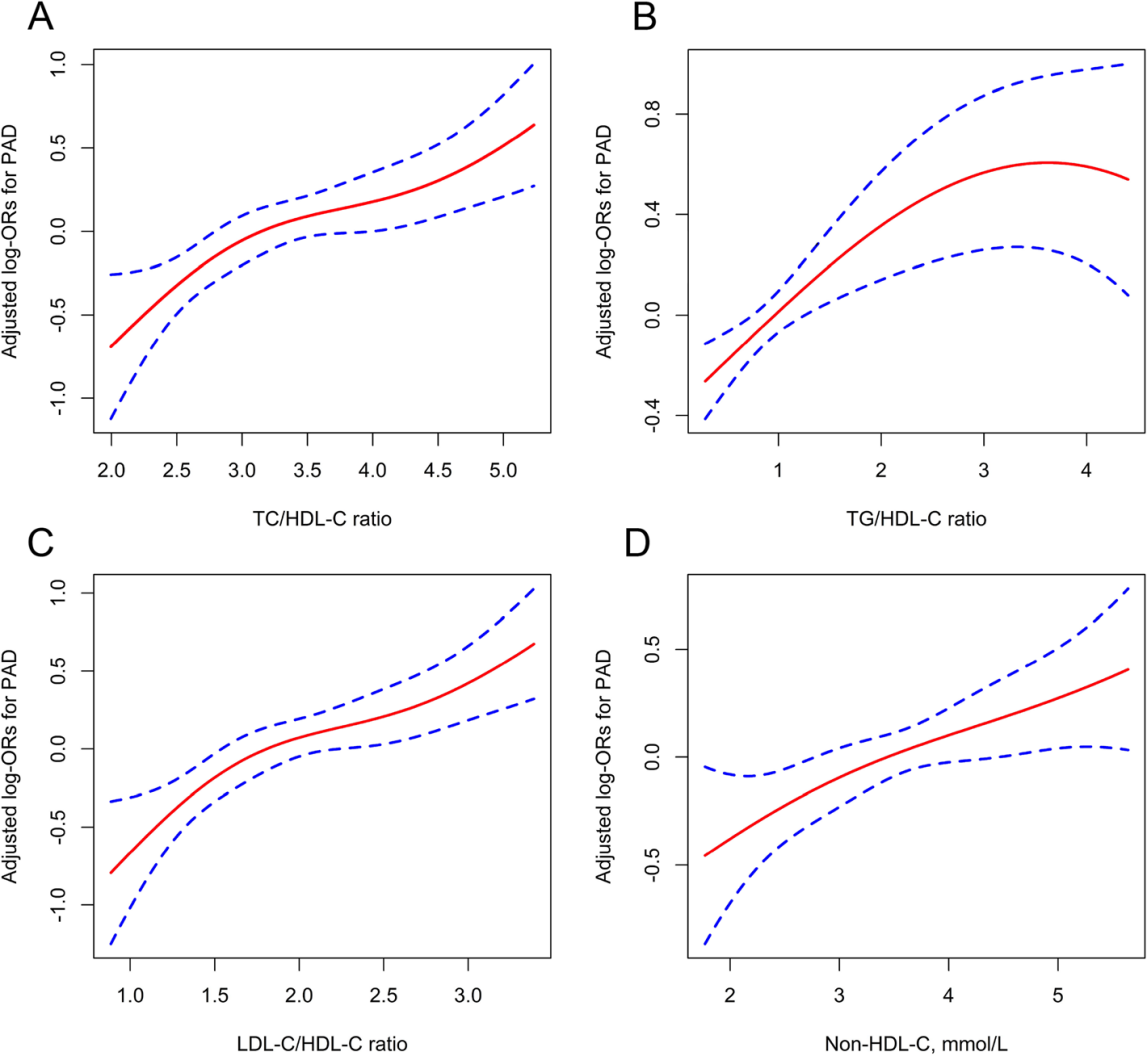
The association between the TC/HDL-C (**a**), TG/HDL-C (**b**), and LDL-C/HDL-C ratios (**c**) and the non-HDL-C (**d**) level and the risk of PAD. The solid line and dashed line represent the estimated values and their corresponding 95% confidence interval, respectively. The adjustment factors included age, sex, BMI, SBP, DBP, smoking status, alcohol drinking statuse, fasting glucose, total homocysteine, eGFR, self-reported diabetes, self-reported stroke, lipoprotein-lowering drugs, and antihypertensive drugs

The association between nontraditional lipid profiles and PAD was assessed using multiple logistic regression analyses, and the results are listed in Table 2. Only the continuous TC/HDL-C and LDL-C/HDL-C ratios were significantly and positively associated with PAD in the crude model. However, after multivariable adjustment, the per SD increment of the TC/HDL-C ratio, TG/HDL-C ratio, LDL-C/HDL-C ratio, and non-HDL-C level were all significantly associated with a 37, 14, 40, and 24% higher risk for PAD, respectively. Consistently, when the nontraditional lipid profiles were assessed as tertiles, the adjusted ORs (95% CI) were 1.77 (1.31, 2.40), 1.71 (1.25, 2.34), 2.03 (1.50, 2.74), and 1.70 (1.25, 2.31) for the top tertiles of the TC/HDL-C, TG/HDL-C, LDL-C/HDL-C ratios, and non-HDL-C, respectively, compared with the lowest tertiles. Additionally, the association between the four nontraditional lipid profiles and PAD was likely to be linear (all *P* for trend < 0.001). Similar trends were found when nontraditional lipid profiles were assessed as quintiles (see Additional file 1: Table S1). Further analyses using restricted cubic spline confirmed the linearly positive association between the four nontraditional lipid profiles and the risk of PAD **(**Fig. 2**)**.

**Table 2:** Odds ratio of PAD according to continuous or tertiles of nontraditional lipid profiles

Variables	N	Events, n(%)	Crude		Model 1	
			OR (95%CI)	***P*** value	OR (95%CI)	***P*** value
TC/HDL-C ratio (Per 1 SD increase)	10,900	310 (2.8%)	1.20 (1.08, 1.34)	< 0.001	1.37 (1.22, 1.54)	< 0.001
Tertiles of TC/HDL-C ratio						
T1(< 3.0)	3633	90 (2.5%)	1.00 (reference)		1.00 (reference)	
T2(3.0–3.7)	3633	103 (2.8%)	1.15 (0.86, 1.53)	0.343	1.36 (1.01, 1.84)	0.045
T3(≥3.7)	3634	117 (3.2%)	1.31 (0.99, 1.73)	0.058	1.77 (1.31, 2.40)	< 0.001
*P* for trend				0.057		< 0.001
TG/HDL-C ratio (Per 1 SD increase)	10,900	310 (2.8%)	0.93 (0.82, 1.06)	0.302	1.14 (1.01, 1.29)	0.029
Tertiles of TG/HDL-C ratio						
T1(< 0.7)	3633	100 (2.8%)	1.00 (reference)		1.00 (reference)	
T2(0.7–1.3)	3633	106 (2.9%)	1.06 (0.80, 1.40)	0.672	1.27 (0.95, 1.70)	0.111
T3(≥1.3)	3634	104 (2.9%)	1.04 (0.79, 1.38)	0.778	1.71 (1.25, 2.34)	< 0.001
*P* for trend				0.779		< 0.001
LDL/HDL-C ratio (Per 1 SD increase)	10,900	310 (2.8%)	1.22 (1.10, 1.36)	< 0.001	1.40 (1.25, 1.57)	< 0.001
Tertiles of LDL/HDL-C ratio						
T1(< 1.7)	3633	91 (2.5%)	1.00 (reference)		1.00 (reference)	
T2(1.7–2.2)	3632	91 (2.5%)	1.00 (0.75, 1.34)	0.999	1.23 (0.91, 1.68)	0.182
T3(≥2.2)	3635	128 (3.5%)	1.42 (1.08, 1.87)	0.012	2.03 (1.50, 2.74)	< 0.001
*P* for trend				0.009		< 0.001
Non-HDL-C (Per 1 SD increase)	10,900	310 (2.8%)	1.08 (0.97, 1.21)	0.163	1.24 (1.10, 1.39)	< 0.001
Tertiles of Non-HDL-C						
T1(< 3.1)	3621	90 (2.5%)	1.00 (reference)		1.00 (reference)	
T2(3.1–3.9)	3633	112 (3.1%)	1.25 (0.94, 1.65)	0.123	1.58 (1.17, 2.12)	0.003
T3(≥3.9)	3646	108 (3.0%)	1.20 (0.90, 1.59)	0.213	1.70 (1.25, 2.31)	< 0.001
*P* for trend				0.223		< 0.001

Model 1 was adjusted for age, sex, BMI, SBP, DBP, smoking status, alcohol drinking status, fasting glucose, total homocysteine, eGFR, self-reported diabetes, self-reported stroke, lipoprotein-lowering drugs, and antihypertensive drugs

Abbreviations: *PAD* Peripheral arterial disease; *OR* Odd ratio; *95% CI* 95% Confidence interval; *TC* Total cholesterol; *TG* Triglyceride; *HDL-C* High-density lipoprotein cholesterol; *LDL-C* Low-density lipoprotein cholesterol; *non-HDL-C* Non-high-density lipoprotein cholesterol

### Subgroup analyses

Subgroup analyses by stratification of the major covariates were performed to further confirm that the findings were reliable in the presence of potential confounders. None of the stratified variables, including sex, age, BMI, smoking, drinking, SBP, tHcy and fasting glucose, significantly modified the association between the four types of nontraditional lipid profiles and the risk of PAD (all *P*-interaction > 0.05) **(**see Additional file 1: Fig. S1–4**)**.

## Discussion

In this sample of large rural residents, the nontraditional lipid profiles were all positively related to the risk of PAD in hypertension participants in China. The restricted cubic spline indicated that the relationship of the four nontraditional lipid indices with PAD was linear. Moreover, the findings suggested that the TC/HDL-C and LDL-C/HDL-C ratios were better early predictors of PAD risk than other nontraditional lipid indices.

To date, the relationship between nontraditional lipid indices and arteriosclerotic cardiovascular disease (ASCVD) has not been comprehensively examined. Lee et al. [11] performed a prospective study of 107 participants with type 2 diabetes in Taiwan and found that TC/HDL-C showed an inverse trend in changing in the ABI (β = − 0.212, 95% CI: − 0.043−− 0.001). Another post hoc data analysis from a randomized controlled trial of 1599 community-based elderly participants reported that the TC/HDL-C ratio (per SD increment, OR: 1.31; 95% CI: 1.14–1.49), non-HDL-C level (OR: 1.15; 95% CI: 1.01–1.31) and LDL-C/HDL-C ratio (OR: 1.25; 95% CI: 1.10–1.43) were significantly correlated with PAD [12]. Moreover, another population-based study of the elderly in rural China also observed a significant association between the LDL-C/HDL-C ratio and PAD (highest compared with the lowest tertile; OR: 2.56; 95% CI, 1.37 to 4.81) [20]. Additionally, Ridker et al. [21] and Pradhan et al. [22] separately reported the results concerning the association between the TC/HDL-C ratio and PAD in male physicians and female health professionals in the United States. Both studies yielded the same results that the TC/HDL-C ratio was significantly associated with PAD. However, Tongdee et al. found that the TC/HDL-C, TG/HDL-C and LDL-C/HDL-C ratios failed to predict early subclinical atherosclerosis in perimenopausal/menopausal women [23]. A cross-sectional survey [24] based on 2982 elderly Beijing residents found that the TG/HDL-C ratio was not associated with a low ABI (ABI ≤ 0.9) (highest compared with the lowest quartile; OR: 1.64; 95% CI, 0.88 to 3.07; *P* ≥ 0.05). This study also showed that the trends of the association between the TC/HDL-C ratio and the risk of PAD were nonlinear. The PAD risk was almost identical on the left side of inflexion and then increased linearly when the TC/HDL-C ratio ≥ 3. However, no studies have assessed the association between nontraditional lipid profiles and PAD in hypertensive populations.

Previous studies did not find that the linear relationship, to some extent, may be attributed to differences in patient characteristics, tHcy levels, or adjustment of confounders. First, the participants in the present study had hypertension, and the study by Lee et al. included only participants with type 2 diabetes. Hypertension may exhibit heterogeneity, and different diseases have different injury saturation point. Second, the lipid levels are significantly different based on region and diet, and the baseline lipid levels may impact the levels of lipid ratios and the prevalence of PAD. The high-carbohydrate diets of Chinese populations may contribute to hypertriglyceridemia, and the standard Western high-fat diets are more likely to induce hypercholesterolemia [25]. The present study was performed in a population with high-carbohydrate diets, and the studies of Ridker et al. and Pradhan et al. were undertaken in regions with the standard high-fat diets. Indeed, obvious geographical differences in PAD incidence also exist in China [26]. The present study was performed in the inland areas of the south, but the studies of Liang et al. and Zhan et al. were conducted in northern areas, while the study by Chi et al. was performed in the coastal areas. Third, the mean baseline tHcy was 18.0 μmol/L in the present study, much higher than normal levels. High tHcy could causes disorders of lipid metabolism and further atherosclerotic disease [27, 28]. Therefore, the association between nontraditional lipid profiles and PAD in a group of low tHcy concentrations cannot be examined. Overall, the current studies are just hypothesis-generating, and further investigations are necessary to consolidate the results of this study.

The exact mechanisms by which the nontraditional lipid indices could predict the risk of PAD were unclear, but using nontraditional lipid indices to predict PAD is biologically plausible. First, significant alterations in nontraditional lipid profiles, which are likely the result of dyslipidaemia in the early stages of PAD, may be characterized as a state of miniaturized low-density lipoprotein particle (LDL-P), and decreased high-density lipoprotein particle (HDL-P). The TC/HDL-C ratio, as a manifestation of atherogenic particle load, was associated with LDL-P, and the particle differences in low-density lipoprotein (LDL) may be attributed to a source of residual risk of cardiovascular events and a unique lipoprotein signature for PAD [29, 30]. In addition, previous studies showed that an elevated serum TG/HDL-C ratio independently predicted a decrease in LDL-P size [31]. Previous studies showed that small dense LDL (sdLDL) promoted pro-atherogenic modifications with the characteristics of incremental flux into the arterial intima, prolonged circulation time and reduced LDL receptor affinity [32, 33]. Moreover, the TC/HDL-C and TG/HDL-C ratios may reflect a decrease in mature large-size HDL-P, which is related to anti-atherosclerosis [25, 34, 35]. Non-HDL-C is a recognized risk factor for ASCVD because of its containment all of the atherogenic lipoproteins, and an epidemiological survey showed that non-HDL-C was a stronger lipid parameter of atherogenesis than LDL-C [15]. Overall, the TC/HDL-C, TG/HDL-C and non-HDL-C ratios indirectly suggest early PAD risk by reflecting the size and density of LDL-P, HDL-P and other atherosclerotic factors, such as chylomicron, very-low density lipoprotein (VLDL), and intermediate density lipoprotein (IDL), which are all closely related to CVD. Second, the TC/HDL-C and TG/HDL-C ratios were strongly related to insulin resistance, whereas insulin resistance was closely related to the development of PAD [36–38]. Third, most participants (98.7%) in the present study had hyperhomocysteinaemia (HHcy, defined as tHcy ≥10 μmol/L) simultaneously, and HHcy may reflect the clinical significance of nontraditional lipid indices by mediating the changes in blood lipid levels and increased adverse effects of lipids on atherosclerosis. In addition to reducing the apolipoprotein A-I composite, HHcy also reduces the high-density lipoprotein (HDL) content in circulation via the promotion of HDL-C clearance [28, 39]. Furthermore, elevated LDL levels in patients with HHcy could be more likely to produce atherosclerotic disease [40, 41]. Therefore, the LDL-C/HDL-C ratio may be a good indicator for identifying the risk of early atherosclerosis, and it can replace the standard lipid profile in the HHcy population. Because a lot of studies have come to the same conclusion that nontraditional lipid profiles would better represent the potential atherosclerotic evolution, using nontraditional lipid profiles to assess the risk of atherosclerotic diseases is reasonable.

### Study strengths and limitations

This study is currently the largest study to assess the association between nontraditional lipid profiles and the risk of PAD in hypertension patients. Nevertheless, several potential limitations of this study are noteworthy. First, this study was a cross-sectional study, which makes it difficult to explain the causal relationship between non-traditional lipid profiles and PAD. Second, the study population comprised rural hypertensive patients in southern China, and the study subjects were over 18 years old. Therefore, these results cannot be generalized to other age groups, regions, or types of diseases.

## Conclusion

Among Chinese hypertensive adults, all of the nontraditional lipid indices (the TC/HDL-C, TG/HDL-C, LDL-C/HDL-C ratios, and non-HDL-C) were independently and positively associated with PAD. In addition, the TC/HDL-C and LDL-C/HDL-C ratios better predicted the risk of PAD than other nontraditional lipid indices. Therefore, the use of these nontraditional lipid profiles, which are inexpensive and easy to calculate in clinical practice, may improve the risk stratification of ASCVD and select favourable candidates for aggressive lipoprotein-lowering therapy.

## Supplementary Information

**Additional file 1: Table S1.** Odds ratio of PAD according to continuous or quintiles of nontraditional lipid profiles. **Fig. S1**. The association between the TC/HDL-C ratio (per SD increment) and the risk of peripheral arterial disease (PAD) in various subgroups*. ^*^Adjusted, if not stratified, for age, sex, BMI, SBP, DBP, smoking status, alcohol drinking status, fasting glucose, total homocysteine, eGFR, self-reported diabetes, self-reported stroke, lipoprotein-lowering drugs, and antihypertensive drugs. History of diabetes was defined as self-reported diabetes, or use of glucose-lowering drugs. **Fig. S2**. The association between the TG/HDL-C ratio (per SD increment) and the risk of peripheral arterial disease (PAD) in various subgroups*. ^*^Adjusted, if not stratified, for age, sex, BMI, SBP, DBP, smoking status, alcohol drinking status, fasting glucose, total homocysteine, eGFR, self-reported diabetes, self-reported stroke, lipoprotein-lowering drugs, and antihypertensive drugs. History of diabetes was defined as self-reported diabetes, or use of glucose-lowering drugs. **Fig. S3**. The association between the LDL-C/HDL-C ratio (per SD increment) and the risk of peripheral arterial disease (PAD) in various subgroups*. ^*^Adjusted, if not stratified, for age, sex, BMI, SBP, DBP, smoking status, alcohol drinking status, fasting glucose, total homocysteine, eGFR, self-reported diabetes, self-reported stroke, lipoprotein-lowering drugs, and antihypertensive drugs. History of diabetes was defined as self-reported diabetes, or use of glucose-lowering drugs. **Fig. S4**. The association between the non-HDL-C (per SD increment) and the risk of peripheral arterial disease (PAD) in various subgroups*. ^*^Adjusted, if not stratified, for age, sex, BMI, SBP, DBP, smoking status, alcohol drinking status, fasting glucose, total homocysteine, eGFR, self-reported diabetes, self-reported stroke, lipoprotein-lowering drugs, and antihypertensive drugs. History of diabetes was defined as self-reported diabetes, or use of glucose-lowering drugs.

## Data Availability

The datasets generated and analysed during the current study are not publicly available because this study is still on-going and the follow-up is not finished, but they are available from the corresponding author on reasonable request.

## Abbreviations

PAD: Peripheral arterial disease
TC: Total cholesterol
TG: Triglyceride
HDL-C: High-density lipoprotein cholesterol
LDL-C: Low-density lipoprotein cholesterol
non-HDL-C: Non-High-density lipoprotein cholesterol
ABI: Ankle-brachial index
SBP: Systolic blood pressure
DBP: Diastolic blood pressure
CVD: Cardiovascular disease
tHcy: Total homocysteine
BMI: Body mass index
eGFR: Estimated glomerular filtration rate
OR: Odds ratio
CI: Confidence interval
ASCVD: Arteriosclerotic cardiovascular disease
LDL-P: Low density lipoprotein particle
HDL-P: High density lipoprotein particle
sdLDL: Small dense LDL
VLDL: Very low density lipoprotein
IDL: Intermediate density lipoprotein
HHcy: Hyperhomocysteinaemia
