# Supplementary material for: Association between nontraditional lipid profiles and peripheral arterial disease in Chinese adults with hypertension
Source: Lipids Health Dis. 2020 Nov 3;19:231. doi: 10.1186/s12944-020-01407-3 (PMC7640397; doi:10.1186/s12944-020-01407-3)
Supplement: Supplementary file 1 — Additional file 1: Table S1. Odds ratio of PAD according to continuous or quintiles of nontraditional lipid profiles. Fig. S1. The association between the TC/HDL-C ratio (per SD increment) and the risk of peripheral arterial disease (PAD) in various subgroups*. *Adjusted, if not stratified, for age, sex, BMI, SBP, DBP, smoking status, alcohol drinking status, fasting glucose, total homocysteine, eGFR, self-reported diabetes, self-reported stroke, lipoprotein-lowering drugs, and antihypertensive drugs. History of diabetes was defined as self-reported diabetes, or use of glucose-lowering drugs. Fig. S2. The association between the TG/HDL-C ratio (per SD increment) and the risk of peripheral arterial disease (PAD) in various subgroups*. *Adjusted, if not stratified, for age, sex, BMI, SBP, DBP, smoking status, alcohol drinking status, fasting glucose, total homocysteine, eGFR, self-reported diabetes, self-reported stroke, lipoprotein-lowering drugs, and antihypertensive drugs. History of diabetes was defined as self-reported diabetes, or use of glucose-lowering drugs. Fig. S3. The association between the LDL-C/HDL-C ratio (per SD increment) and the risk of peripheral arterial disease (PAD) in various subgroups*. *Adjusted, if not stratified, for age, sex, BMI, SBP, DBP, smoking status, alcohol drinking status, fasting glucose, total homocysteine, eGFR, self-reported diabetes, self-reported stroke, lipoprotein-lowering drugs, and antihypertensive drugs. History of diabetes was defined as self-reported diabetes, or use of glucose-lowering drugs. Fig. S4. The association between the non-HDL-C (per SD increment) and the risk of peripheral arterial disease (PAD) in various subgroups*. *Adjusted, if not stratified, for age, sex, BMI, SBP, DBP, smoking status, alcohol drinking status, fasting glucose, total homocysteine, eGFR, self-reported diabetes, self-reported stroke, lipoprotein-lowering drugs, and antihypertensive drugs. History of diabetes was defined as self-reported [file 12944_2020_1407_MOESM1_ESM.doc]

**Table S1. Odds ratio of PAD according to continuous or quintiles of nontraditional lipid profiles**

| Variables | OR (95% CI) by quintiles of nontraditional lipid profiles | | | | | | OR (95% CI) per SD increase of lipid profiles | *P* value |
| --- | --- | --- | --- | --- | --- | --- | --- | --- |
|  | Quintile 1 | Quintile 2 | Quintile 3 | Quintile 4 | Quintile 5 | *P* for trend |  |  |
| TC/HDL-C ratio | <2.6 | 2.6-3.1 | 3.1-3.5 | 3.5-4.1 | ≥4.1 |  |  |  |
| Events, n/N (%) | 47//2180 (2.2%) | 59/2180 (2.7%) | 65/2180 (3.0%) | 63/2180 (2.9%) | 76/2180 (3.5%) |  |  |  |
| Model 1 | 1.00 (reference) | 1.26 (0.86, 1.86) | 1.39 (0.95, 2.04) | 1.35 (0.92, 1.98) | 1.64 (1.13, 2.37) | 0.012 | 1.20 (1.08, 1.34) | <0.001 |
| Model 2 | 1.00 (reference) | 1.44 (0.97, 2.13) | 1.73 (1.18, 2.55) | 1.82 (1.23, 2.69) | 2.43 (1.67, 3.55) | <0.001 | 1.37 (1.23, 1.53) | <0.001 |
| Model 3 | 1.00 (reference) | 1.42 (0.95, 2.12) | 1.71 (1.15, 2.53) | 1.85 (1.23, 2.77) | 2.38 (1.60, 3.55) | <0.001 | 1.37 (1.22, 1.54) | <0.001 |
| TG/HDL-C ratio | <0.5 | 0.5-0.8 | 0.8-1.1 | 1.1-1.7 | ≥1.7 |  |  |  |
| Events, n/N (%) | 51/2180 (2.3%) | 66/2180 (3.0%) | 70/2180 (3.2%) | 65/2180 (3.0%) | 58/2180 (2.7%) |  |  |  |
| Model 1 | 1.00 (reference) | 1.30 (0.90, 1.89) | 1.38 (0.96, 2.00) | 1.28 (0.88, 1.86) | 1.14 (0.78, 1.67) | 0.596 | 0.93 (0.82, 1.06) | 0.302 |
| Model 2 | 1.00 (reference) | 1.41 (0.96, 2.05) | 1.84 (1.27, 2.68) | 1.96 (1.34, 2.88) | 2.14 (1.44, 3.18) | <0.001 | 1.14 (1.02, 1.27) | 0.021 |
| Model 3 | 1.00 (reference) | 1.27 (0.87, 1.87) | 1.63 (1.11, 2.40) | 1.81 (1.21, 2.70) | 2.01 (1.32, 3.07) | <0.001 | 1.14 (1.01, 1.29) | 0.029 |
| LDL-C/HDL-C ratio | <1.4 | 1.4-1.8 | 1.8-2.1 | 2.1-2.5 | ≥2.5 |  |  |  |
| Events, n/N (%) | 44/2180 (2.0%) | 70/2180 (3.2%) | 51/2180 (2.3%) | 71/2179 (3.3%) | 74/2181 (3.4%) |  |  |  |
| Model 1 | 1.00 (reference) | 1.61 (1.10, 2.36) | 1.16 (0.77, 1.75) | 1.64 (1.12, 2.39) | 1.70 (1.17, 2.49) | 0.013 | 1.22 (1.10, 1.36) | <0.001 |
| Model 2 | 1.00 (reference) | 1.88 (1.28, 2.78) | 1.54 (1.02, 2.33) | 2.32 (1.57, 3.42) | 2.65 (1.80, 3.91) | <0.001 | 1.41 (1.26, 1.56) | <0.001 |
| Model 3 | 1.00 (reference) | 1.91 (1.29, 2.83) | 1.55 (1.01, 2.37) | 2.36 (1.57, 3.54) | 2.63 (1.74, 3.96) | <0.001 | 1.40 (1.25, 1.57) | <0.001 |
| Non-HDL-C, mmol/L | <2.7 | 2.7-3.3 | 3.3-3.7 | 3.7-4.3 | ≥4.3 |  |  |  |
| Events, n/N (%) | 55/2174 (2.5%) | 54/2180 (2.5%) | 71/2186 (3.2%) | 65/2162 (3.0%) | 65/2198 (3.0%) |  |  |  |
| Model 1 | 1.00 (reference) | 0.98 (0.67, 1.43) | 1.29 (0.90, 1.85) | 1.19 (0.83, 1.72) | 1.17 (0.82, 1.69) | 0.220 | 1.08 (0.97, 1.21) | 0.163 |
| Model 2 | 1.00 (reference) | 1.13 (0.77, 1.66) | 1.65 (1.15, 2.38) | 1.59 (1.09, 2.31) | 1.72 (1.18, 2.50) | <0.001 | 1.23 (1.10, 1.37) | <0.001 |
| Model 3 | 1.00 (reference) | 1.19 (0.80, 1.76) | 1.78 (1.22, 2.59) | 1.81 (1.23, 2.66) | 1.81 (1.22, 2.69) | <0.001 | 1.24 (1.10, 1.39) | <0.001 |

Model 1: unadjusted; Model 2: adjusted for age and sex; Model 3: adjusted for age, sex, BMI, SBP, DBP, smoking status, alcohol drinking status, fasting glucose, total homocysteine, eGFR, self-reported diabetes, self-reported stroke, lipoprotein-lowering drugs, and antihypertensive drugs.

Abbreviations: PAD, peripheral arterial disease; OR, odd ratio; 95% CI, 95% confidence interval; TC, total cholesterol; TG, triglyceride; HDL-C, high-density lipoprotein cholesterol; LDL-C, low-density lipoprotein cholesterol; non-HDL-C, non-high-density lipoprotein cholesterol.


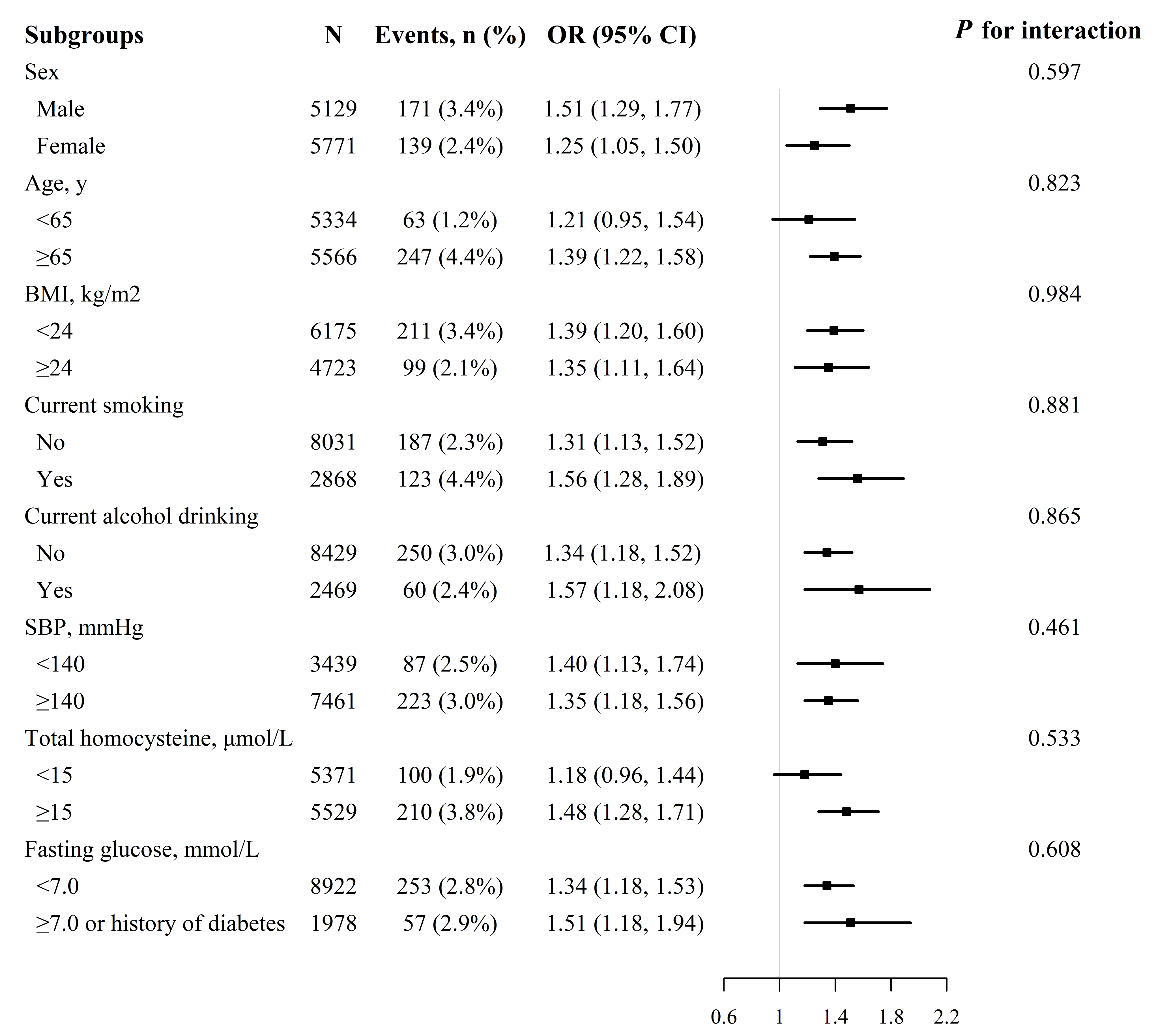


**Fig. S1. The association between TC/HDL-C ratio (per SD increment) and the risk of peripheral arterial disease (PAD) in various subgroups*.**

^*^Adjusted, if not stratified, for age, sex, BMI, SBP, DBP, smoking status, alcohol drinking status, fasting glucose, total homocysteine, eGFR, self-reported diabetes, self-reported stroke, lipoprotein-lowering drugs, and antihypertensive drugs. History of diabetes was defined as self-reported diabetes, or use of glucose-lowering drugs.


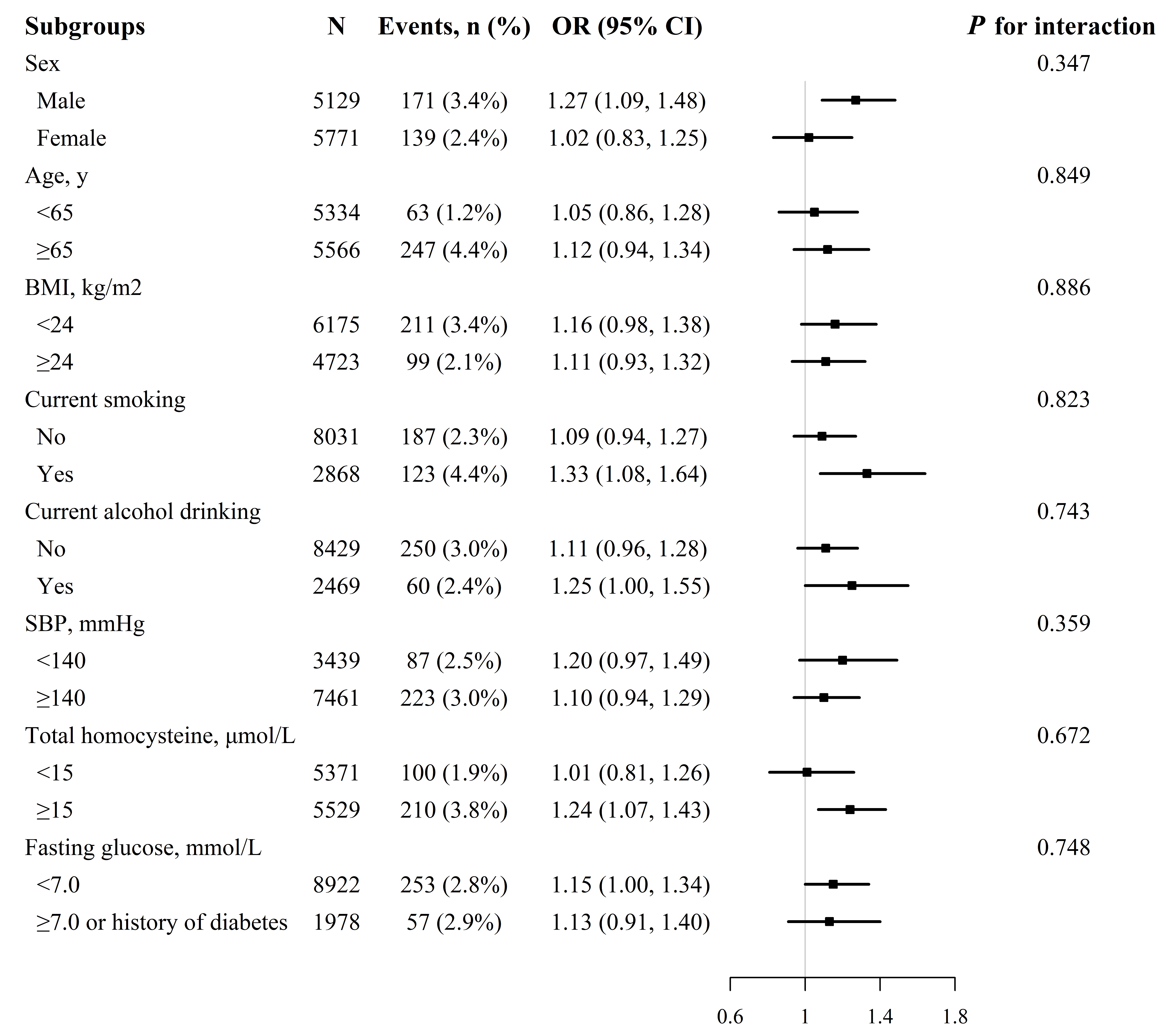


**Fig. S2. The association between TG/HDL-C ratio (per SD increment) and the risk of peripheral arterial disease (PAD) in various subgroups^*^.**

^*^Adjusted, if not stratified, for age, sex, BMI, SBP, DBP, smoking status, alcohol drinking status, fasting glucose, total homocysteine, eGFR, self-reported diabetes, self-reported stroke, lipoprotein-lowering drugs, and antihypertensive drugs. History of diabetes was defined as self-reported diabetes, or use of glucose-lowering drugs.


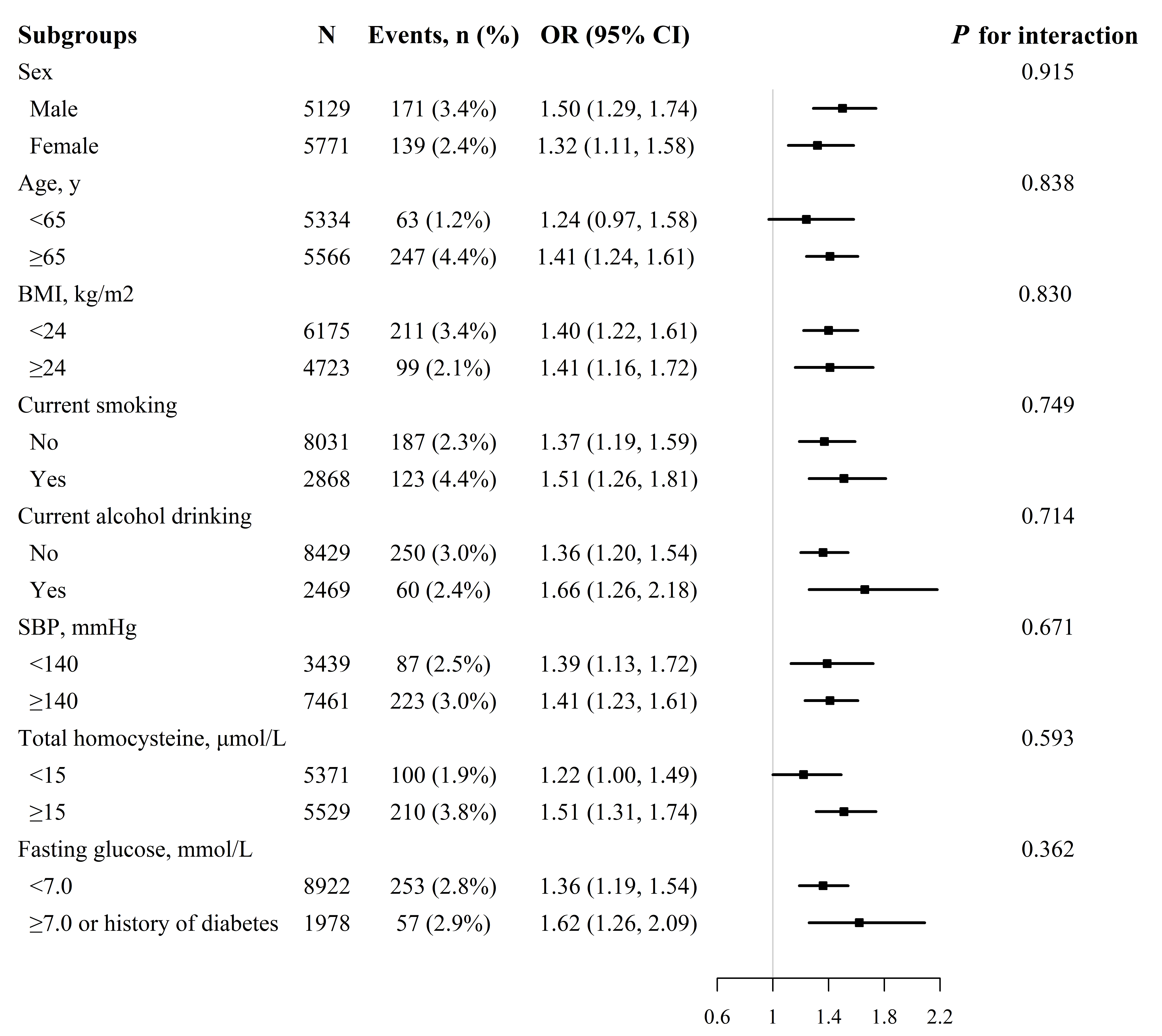


**Fig. S3. The association between LDL-C/HDL-C ratio (per SD increment) and the risk of peripheral arterial disease (PAD) in various subgroups^*^.**

^*^Adjusted, if not stratified, for age, sex, BMI, SBP, DBP, smoking status, alcohol drinking status, fasting glucose, total homocysteine, eGFR, self-reported diabetes, self-reported stroke, lipoprotein-lowering drugs, and antihypertensive drugs. History of diabetes was defined as self-reported diabetes, or use of glucose-lowering drugs.


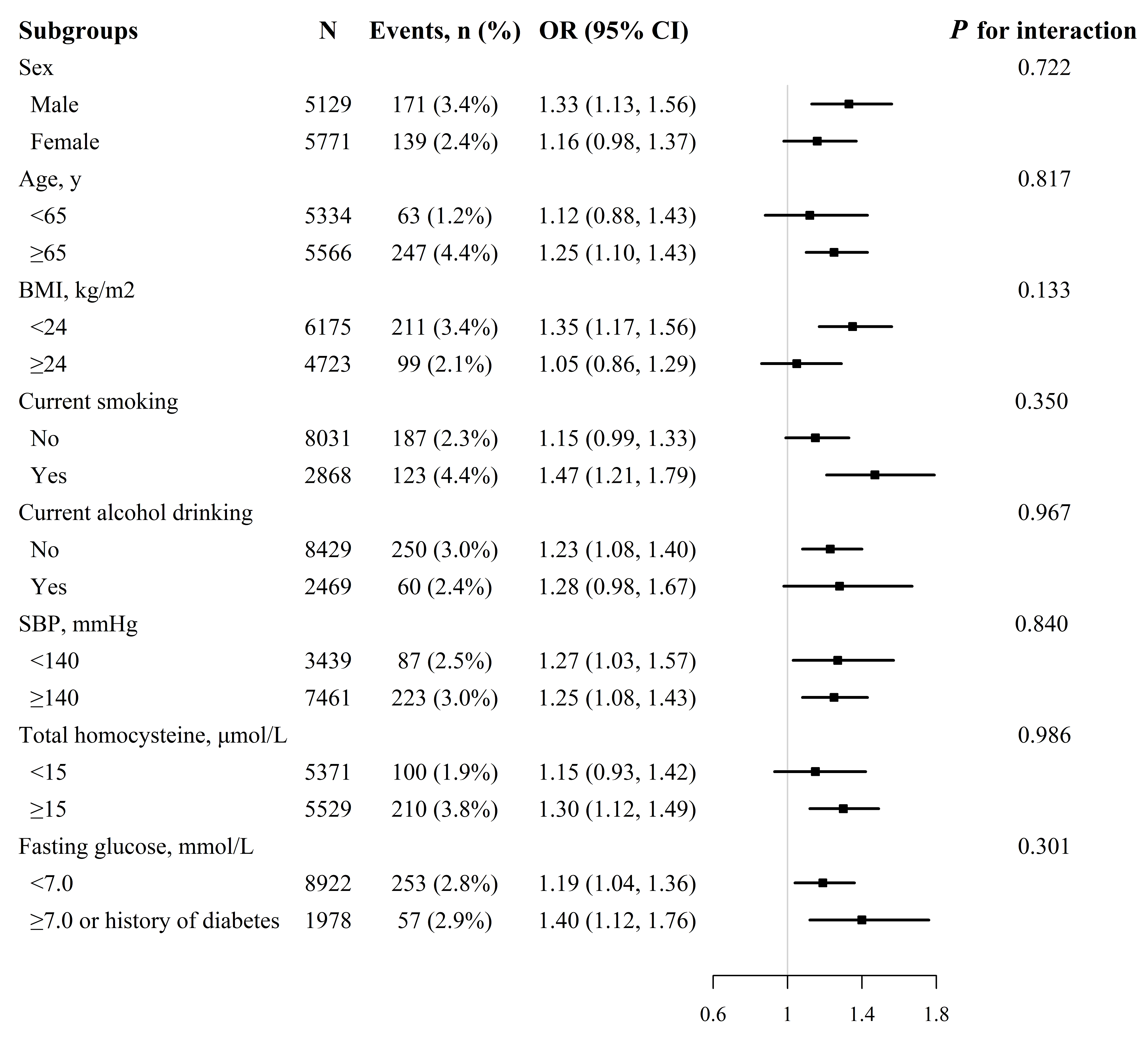


**Fig. S4. The association between non-HDL-C (per SD increment) and the risk of peripheral arterial disease (PAD) in various subgroups^*^.**

^*^Adjusted, if not stratified, for age, sex, BMI, SBP, DBP, smoking status, alcohol drinking status, fasting glucose, total homocysteine, eGFR, self-reported diabetes, self-reported stroke, lipoprotein-lowering drugs, and antihypertensive drugs. History of diabetes was defined as self-reported diabetes, or use of glucose-lowering drugs.
